# Characterization of 3D-Printed Glass Fiber-Filled and Calcium Carbonate-Filled Polypropylene Components for Surgical Planning

**DOI:** 10.3390/polym17192684

**Published:** 2025-10-04

**Authors:** Núria Adell-Gómez, Irene Buj-Corral, Miquel Domingo-Espin, Jordi Llumà, J. Antonio Travieso-Rodríguez, Josep Rubio-Palau, César García-Fontecha, Alejandro Domínguez-Fernández, Arnau Valls-Esteve

**Affiliations:** 1 3D4H Unit, Hospital Sant Joan de Déu, Santa Rosa 39-57, 08950 Esplugues de Llobregat, Spain; nuria.adell@sjd.es (N.A.-G.); arnau.valls@sjd.es (A.V.-E.); 2 TECNOFAB Research Group, Barcelona School of Industrial Engineering (ETSEIB), Universitat Politècnica de Catalunya (UPC), Av. Diagonal, 647, 08028 Barcelona, Spain; alejandro.dominguez-fernandez@upc.edu; 3 Advanced Manufacturing Systems Unit, Eurecat—Technology Centre of Catalonia, Av. Universitat Autònoma, 23, Cerdanyola del Vallès, 08290 Barcelona, Spain; miquel.domingo@eurecat.org; 4 TECNOFAB Research Group, Barcelona East School of Engineering (EEBE), Universitat Politècnica de Catalunya (UPC), Av. Eduard Maristany, 16, 08019 Barcelona, Spain; jordi.lluma@upc.edu (J.L.); antonio.travieso@upc.edu (J.A.T.-R.); 5 Maxillofacial Surgery Department, Hospital Sant Joan de Déu, Santa Rosa 39-57, 08950 Esplugues de Llobregat, Spain; josep.rubio@sjd.es; 6 Orthopedic Surgery Department, Hospital Sant Joan de Déu, Santa Rosa 39-57, 08950 Esplugues de Llobregat, Spain; cesar.garciafontecha@sjd.es

**Keywords:** FFF, FDM, GF-PP, CaCO_3_-PP, dimensional accuracy, roughness, porosity, tensile strength

## Abstract

The purpose of this study is to characterize two different 3D-printed materials, glass fiber-filled polypropylene (GF-PP) and calcium carbonate-filled polypropylene (CaCO_3_-PP), which make it possible to obtain surgical bone models at a reasonable cost. The methodology involved selecting two filaments, among six, which showed better processability in the fused filament fabrication (FFF) process. Then, samples of the two selected materials were 3D printed, followed by characterization in terms of dimensional error, porosity, surface roughness, and mechanical strength. The results showed that both materials can be sterilized, with an increase in dimensional error and porosity after sterilization and slight changes in roughness and tensile strength. Additionally, anatomical models of mandible and femur bones were clinically validated by surgeons.

## 1. Introduction

Additive manufacturing (AM) is a set of technologies that build objects by adding material layer by layer to obtain the desired geometry. There are seven groups of technologies [1], which include Material Extrusion (MEX), Vat Photopolymerization (VPP), Material Jetting (MJ), Powder Bed Fusion (PBD), and Binder Jetting (BJ), among others. Fused filament fabrication (FFF), also known as fused deposition modeling (FDM), is one of the most widely used 3D printing technologies. It is based on extruding a thermoplastic filament through a heated extrusion nozzle, which melts the filament and deposits it to build 3D objects layer by layer.

In recent years, AM has become a powerful tool in the surgical field [2,3]. The ability to create accurate and realistic models of a patient’s anatomy as well as personalized surgical guides has greatly improved the diagnosis and preparation of complex surgeries, allowing surgeons to better plan their surgical approach. AM is being used to create pre-surgical models of bones and other organs, which can be used to simulate surgical techniques and train students’ abilities. Among surgical specialties, 3D printing has become an essential tool in orthopedic and maxillofacial surgery. The capability to print a replica of the patient’s femur or skull, for example, allows the surgeon to plan and practice complex procedures before surgery. This can greatly improve surgical outcomes, as well as reduce the need for invasive procedures or multiple operations. Furthermore, the use of 3D-printed models allows surgeons to explain the pathology and the planned intervention to the patient and their family, improving communication, patient understanding, and acceptance of the treatment [4,5].

Although a common material used in 3D printing for anatomical models, polylactic acid (PLA) has some limitations: it is relatively brittle at room temperature [6], and its fracture properties depend greatly on crystallinity and loading rate [7], which makes it difficult to replicate the properties of bone during drilling, milling, or other common surgical procedures such as plate fixation or Kirschner wire placement. In addition, PLA has a relatively low glass transition temperature (~60 °C), which makes it susceptible to softening or deformation during sterilization processes (e.g., autoclaving at 121 °C), thereby limiting its reusability in sterile surgical environments [8]. As for Acrylonitrile Butadiene Styrene (ABS), it is relatively rigid compared with soft tissues, with an average Young’s modulus of 2394.81 MPa for ABS 3D-printed parts at 100% infill rate [9]. However, it does not accurately replicate the cutting behavior of bone, as it can delaminate between layers during cutting operations [10], and it may crack or fracture under repeated machining [11].

In recent years, solutions specifically tailored for the medical sector have emerged on the market [3]. Notable examples include Stratasys^®^ systems such as the PolyJet J750 Digital Anatomy, and the J5 MediJet Printer, which utilize resin-based materials capable of producing realistic 3D-printed anatomical models that mimic the feel of bone and other tissues [12]. However, there is a lack of solutions that replicate the mechanical properties of bone at a reasonable price. The main challenge in applying 3D printing technologies to anatomical models lies in developing solutions that more accurately mimic tissue behavior at lower prices. In addition, since anatomical models are intended for use in the operating room, materials must withstand sterilization processes involving high temperatures and pressures, which can alter the properties and geometry of the 3D-printed models, thereby compromising their accuracy [13]. There are two main methods to carry out the sterilization procedure for 3D-printed models in a hospital setting [14]: 1. thermal sterilization, using either dry heat or steam, which is also referred to as moist heat sterilization or autoclave; 2. low-temperature sterilization, which involves chemical means (using ethylene oxide or hydrogen peroxide) or radiation (including ionizing or ultraviolet radiation). Polypropylene (PP) is known to have high tolerance to sterilization methods that do not involve elevated temperatures, such as ethylene oxide sterilization, with minimal alteration of its mechanical properties. Nevertheless, autoclaving is also recommended, particularly for single sterilization cycles [15]. On the other hand, irradiated PP is more susceptible to biodegradation than autoclaving [16].

**Figure 1:**
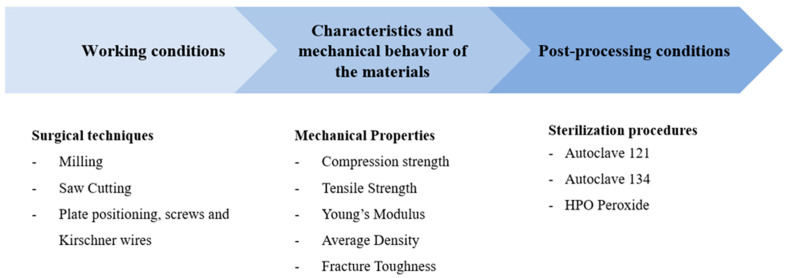
Key factors in selecting 3D-printed materials for bone mimicry.

The key factors that have been identified to consider when developing new materials for bone mimicry in a hospital setting are summarized in Figure 1.

Table 1 shows the properties of bone [17,18,19]. Bone consists of two main tissue subtypes: cortical bone and trabecular bone. It should be noted that the reported values may vary depending on the age and the methods used to assess the bone samples [20].

In Table 2, a comparison of the mechanical properties of different FFF materials is presented.

Table 1 shows that the maximum compressive strength of cortical bone is higher than its tensile strength. For trabecular bone, the ranges of compressive and tensile strength overlap. The maximum strength and Young’s modulus are higher for cortical bone than for trabecular bone.

Some authors have studied the performance of different materials for use as surgical bone models. For example, McMillan et al. [21] found that white photo-crosslinkable acrylic resin produced via stereolithography (SLA) performed better in drilling processes than FDM 3D-printed ABS. However, there is still little information in the literature regarding the mechanical and dimensional properties of composite materials for use in surgical models, as well as their haptic performance during machining and their ability to replicate bone anatomy. PP is known to provide high biocompatibility in implants [22]. This study investigates the feasibility of PP-based composites as an affordable material for surgical models that can enhance the haptic feedback experienced by surgeons in training, compared with currently available FDM filaments. These materials are not required to fully replicate the behavior of bone, but should provide sufficient mechanical stability for their intended applications, such as temporary fixation, intraoperative handling, and anatomical training models.

Table 2 shows that the tensile strength of PP used as a base material is approximately 15 MPa. However, when filled with glass fiber the maximum tensile strength can increase to 50 MPa [23]. In the case of calcium carbonate-filled PP, although the yield stress decreases slightly with filler content, the elastic modulus can rise to over 4000 MPa [24]. Although these models are not intended for implantation and therefore do not affect the correct positioning of an implant, it is advisable that their dimensions closely match those of real bone to ensure accurate surgical rehearsal. In this work, two different materials —glass fiber-filled polypropylene (GF-PP) and calcium carbonate-filled polypropylene (CaCO_3_-PP)— are characterized in terms of dimensional accuracy and tensile strength. The base material, PP, has a relatively high glass transition temperature of approximately 160 °C. For this reason, the material is expected to exhibit low deformation during sterilization processes. Glass fiber is known to increase the stiffness of polymeric materials, while calcium carbonate is sometimes employed as a wax-like additive in polymers. Both materials are expected to enhance the performance of PP during machining of surgical models, without compromising other properties such as mechanical strength, dimensional accuracy, porosity, or surface roughness.

## 2. Materials and Methods

The methodology followed in this study comprised several steps: 3D printing of the specimens, sterilization, clinical validation, and the characterization of the samples in terms of dimensional error, density, porosity, surface roughness, and tensile strength.

### 2.1. Material Preparation

Six new filaments were produced, containing either PP with glass fiber (GF) or PP with calcium carbonate (CaCO_3_). The first group of four materials was produced by mixing THERMOFIL AX93879 (Sumika Polymer Compounds America LLC, Farmington Hills, MI), a PP material containing 20% GF, and diluting it with PP Hifax EP3080 YF10-52-101M (LyondellBasell Industries, Amsterdam, The Netherlands) to produce filaments with lower GF contents of 5%, 8%, 10%, and 15%. The second group of two materials was produced using the same PP matrix to dilute the GF while adding CaCO_3_ (IP-32I CaCO_3_ Filler Compound/Masterbatch from Ecoplast, S.L., Montmeló, Spain) at different quantities (35% and 55%). Reinforcements were introduced via masterbatch formulations. All filaments had a diameter of 2.85 ± 0.15 mm and a rough surface.

**Figure 2:**
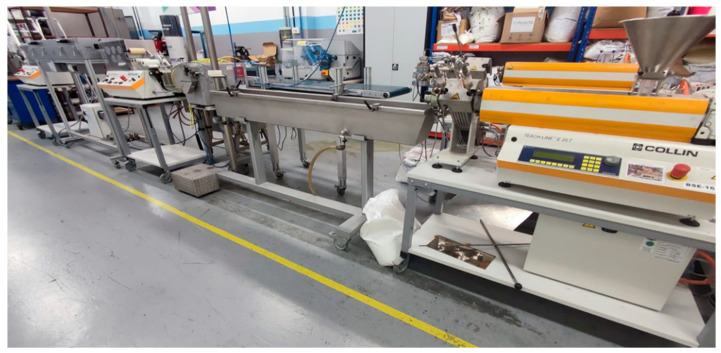
Filament production line using the COLLIN TEACH-LINE E20T single-screw extruder.

All six filaments (PP + 5% GF, PP + 8% GF, PP + GF 10%, PP + GF 15%, PP+ 35% CaCO_3_, and PP + 35% CaCO_3_) were manufactured using the COLLIN TEACH-LINE E20T single-screw extruder (COLLIN Lab & Pilot Solutions GmbH, Maitenbeth, Germany) along with a water bath for cooling, stretching rollers, an oven, and a winding roller (Figure 2).

For PP/CaCO_3_ blends, melt temperatures ranged from 179 °C to 190 °C, with screw speeds between 20 and 37 min^−1^, and tailored temperature profiles across seven heating zones. PP/GF blends required higher melt temperatures (206 °C to 209 °C) and screw speeds of 36 to 40 min^−1^ to stabilize the filament diameter within the target range of 2.85 ± 0.15 mm. Despite achieving dimensional consistency, PP/GF filaments exhibited a rough surface texture. Complete recording of extrusion parameters—including pump rates, module speeds, and zone temperatures—is critical for correlating processing conditions with filament quality and mechanical performance.

Among the six materials studied, PP + GF 5% and PP + CaCO_3_ 35% were selected because they provided the best printability in preliminary tests, allowing 3D printing without noticeable defects, excessive difficulty, or performance loss. In contrast, filaments with higher additive content were difficult to coil and prone to breakage.

### 2.2. Determination of the Melt Flow Index

To evaluate the influence of additive content on the rheological behavior of polypropylene (PP) composites, the Melt Flow Index (MFI) was measured for the different formulations. The MFI is defined as the mass of polymer, in grams, that flows through a capillary of specific diameter and length in ten minutes.

(1) $$MFI=\frac{600s}{t}\times M\;(g/10\;\min)$$

where t = time of extrudate in s, and M is the mass of extrudate in g.

The test was carried out under the ISO 1133 standard [25], using the typical test conditions for polypropylenes (230 °C/2.16 kg). A Modular Flow Index CEAST MF30 (Instron, Norwood, MA, USA) apparatus was used to determine the MFI.

The molecular weight (Mw) of each blend was estimated using an empirical inverse power law relationship (Equation (2)):

(2) $$Mw=K\cdot {MFI}^{-n}\;(g/\mathrm{mol})$$

where (K = 1.2 × 10^5^) and (n = 0.5), values commonly used for PP homopolymers [26]. This approach assumes that higher MFI values correspond to lower molecular weights due to reduced chain entanglement and viscosity.

### 2.3. Three-Dimensional Printing of the Samples

**Figure 3:**
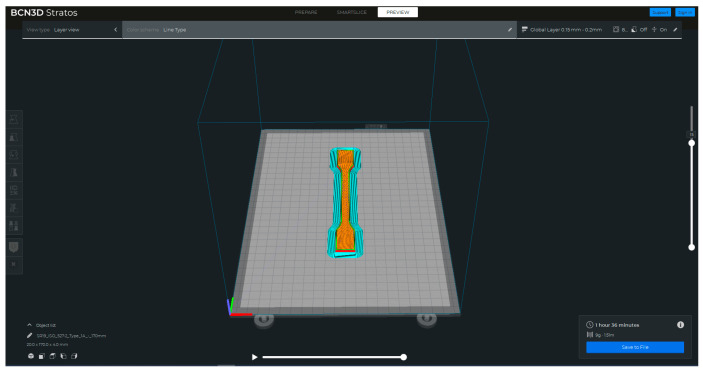
Preview of the printing process on BCN3D Stratos software.

The samples were 3D printed using an FFF 3D printer, the Sigma R19 from BCN3D Technologies (Gavà, Spain). These samples were in the form of tensile specimens according to the ISO527 type IA standard [27] (Figure 3). The nominal dimensions of the samples were 4 mm in thickness and 10 mm in width at the narrow section. The Stratos software (v 2.0.0) (BCN3D Technologies, Gavà, Spain) was used for slicing the geometry of the part.

The 3D printing conditions for the two selected filaments are summarized in Table 3. These conditions included various parameters such as nozzle diameter, layer height, and printing and build plate temperature, among others. A total of 20 samples were 3D printed for each filament.

**Figure 4:**
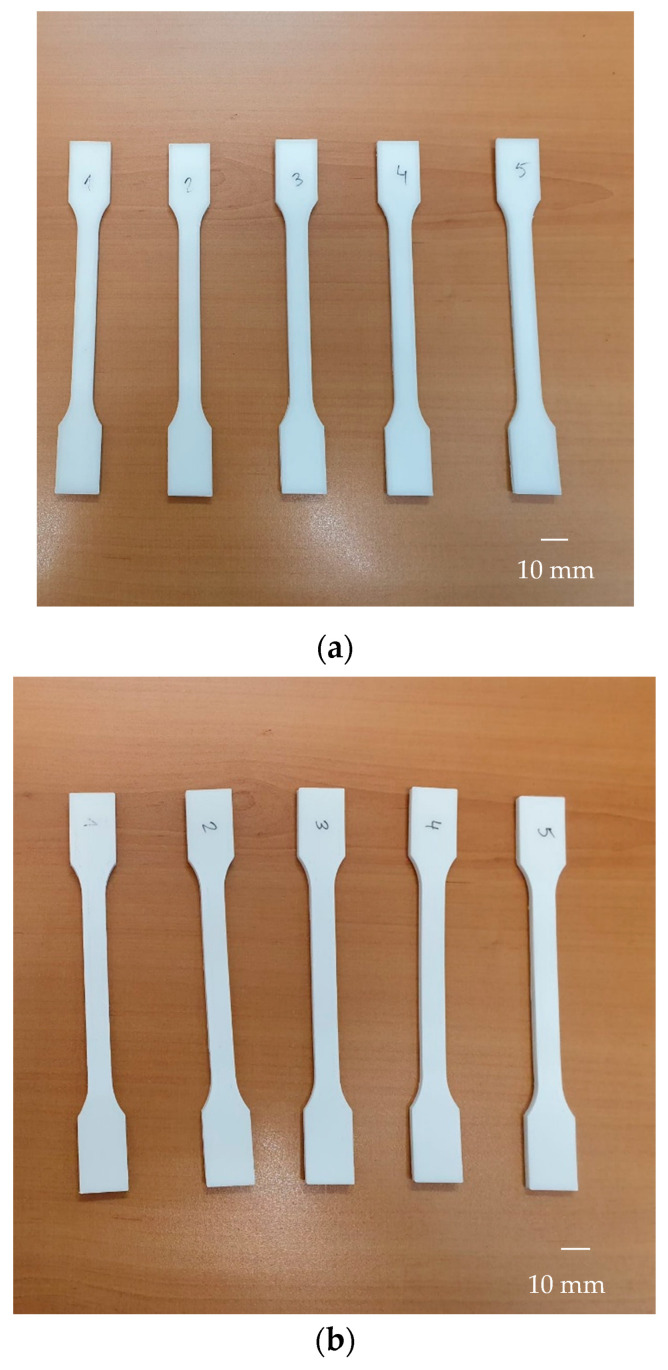
Tensile specimens for (**a**) PP GF 5% and (**b**) PP + CaCO_3_ 35%.

Among the 20 samples for each condition, five were selected as control samples and were randomly labeled 1–5. Figure 4 shows the control samples for PP + GF 5% (Figure 4a) and PP + CaCO_3_ 35% respectively (Figure 4b).

For the clinical validation, 2 mandible and 2 femur models were 3D printed using the same materials and printing conditions, except for the infill rate, which was set to 5% with a grid pattern.

### 2.4. Sterilization of the Samples

The remaining 15 of 20 tensile strength samples for each material underwent sterilization procedures, using Matachana^®^ machines (Antonio Matachana, S.A., Castelldefels, Spain). The sample size was determined based on statistical significance, previous experience, and a review of the literature. The distribution of the sample groups and the respective sterilization procedures applied are as follows:

- Group 1 (control): This group consisted of five tensile specimens that did not undergo any sterilization or disinfection procedure and served as the control group.
- Group 2 (hydrogen peroxide HPO): A total of five tensile specimens were subjected to sterilization using the hydrogen peroxide (HPO) method. This chemical sterilization process involved disinfection using water vapor saturated with hydrogen peroxide. Operating at a temperature of 60 °C and a pressure of 692 hPa, the process effectively eliminates a broad spectrum of microorganisms. Unlike other methods, hydrogen peroxide sterilization does not require high pressure. The entire process was completed within approximately 50 min.
- Group 3 (Autoclave 121): Another group of five tensile specimens were sterilized using the Autoclave 121 method. This thermal sterilization process involves subjecting the specimens to high-temperature and -pressure conditions. The temperature inside the autoclave is maintained at 121 °C, while the pressure remains at 1 atm (101.325 Pa). High pressure is crucial for raising the boiling point of water, ensuring the uniform application of temperature and pressure to all materials within the autoclave. The sterilization process lasted approximately 55 min.
- Group 4 (Autoclave 134): The remaining five tensile specimens were sterilized using the Autoclave 134 method. This sterilization process is similar to Autoclave 121 but operates at a higher temperature of 134 °C and a pressure of 2 atm (202.650 Pa). Additionally, the processing time is shorter, lasting approximately 40 min.

By categorizing the samples into different groups and subjecting them to various sterilization procedures, a comprehensive evaluation of the effects of sterilization on the printed samples was conducted. This approach allows for a comparative analysis of the different sterilization methods and their impact on the materials under investigation.

### 2.5. Characterization of the Samples

To comprehensively assess the characteristics of all tensile test samples, including both the sterilized groups and the control groups, several parameters were analyzed, namely dimensional error, porosity, and roughness. The methodologies are explained in the following subsections. For each characteristic, the distributions of two groups at a time was compared by means of the Kolmogorov–Smirnov test with α = 0.05.

#### 2.5.1. Dimensional Error

The sample dimensions were measured using a digital micrometer (Mitutoyo, Kawasaki, Japan). The relative thickness error was calculated to evaluate deviations from the nominal dimension of 4 mm.

#### 2.5.2. Porosity

Samples were weighted using a Kern 440-33N scale (Kern & Sohn GmbH, Balingen, Germany) with a precision of 0.01 g. Porosity was calculated by considering the density of the solid filament and comparing it with the overall density of the samples.

#### 2.5.3. Roughness

**Figure 5:**
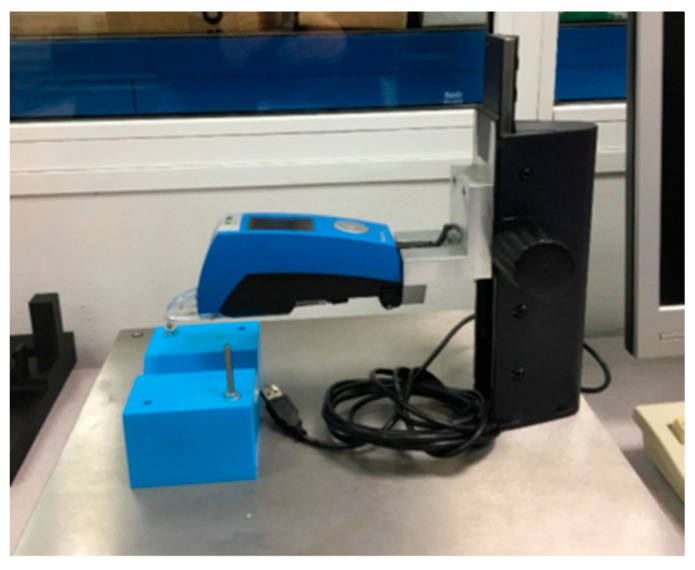
Hommel Etamic W5 roughness meter.

Surface roughness was measured using a contact Hommel Etamic W5 roughness meter (Hommel Etamic, Villingen-Schwenningen, Germany) (Figure 5).

Measurements were taken on the lateral surface of the samples, which is perpendicular to the material layers. A Gaussian filter with a cut-off length of 0.8 mm was applied to ensure accurate measurements, and the total evaluation length was 4.8 mm.

#### 2.5.4. Mechanical Strength

**Figure 6:**
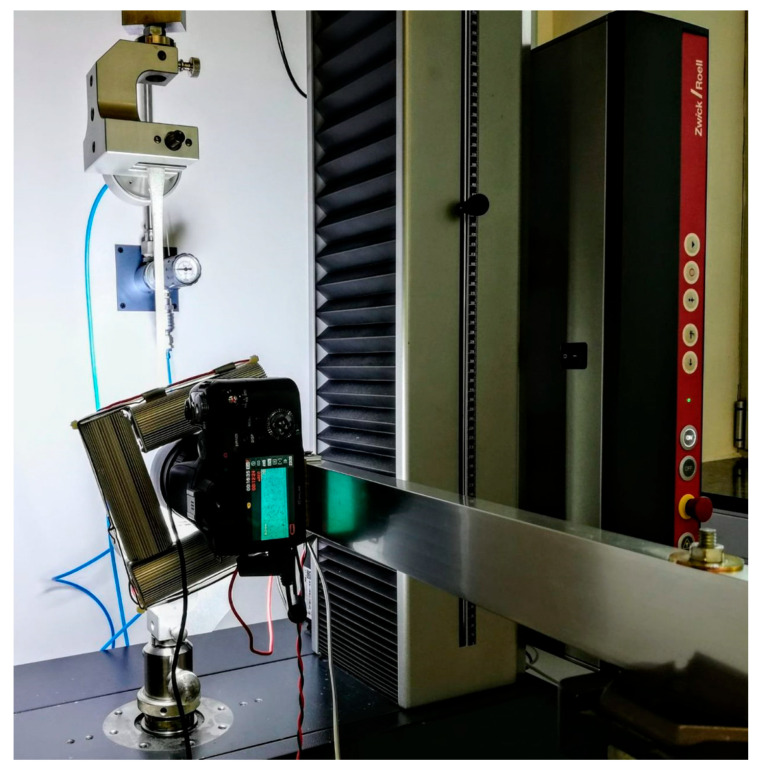
Set-up of the mechanical test using the Zwick All-round Z005 universal testing machine.

Mechanical tests were performed to determine the maximum tensile strength of both the sterilized and control specimens (Figure 6).

Tests were performed using a Zwick All-round Z005 universal testing machine (Ulm, Germany) with a maximum force of 5 kN. The tests were carried out in displacement control mode at a speed of 5 mm/min. All tests were conducted under laboratory conditions (20–25 °C and 30–60% humidity).

### 2.6. Clinical Validation

The validation was carried out exclusively by two senior specialists (heads of the maxillofacial surgery and orthopedic surgery departments) at Sant Joan de Déu Barcelona Children’s Hospital, Esplugues de Llobregat, Spain. The methodological decision to include only these two evaluators is based on several considerations:

Level of expertise: Both surgeons have extensive clinical and academic backgrounds. Their expert judgment ensures a highly qualified and standardized assessment of model fidelity, minimizing potential biases associated with variability in the learning curve.

Exploratory/pilot design: This study was conceived as an initial validation of the feasibility and accuracy of 3D-printed bone models. At this preliminary stage, the primary goal was to demonstrate the reproducibility of the material and anatomical fidelity of the prints, rather than to investigate interobserver variability in a broader cohort. The inclusion of highly specialized experts was therefore the most appropriate strategy for this first step. Future studies will include a larger and more diverse group of clinicians, allowing the exploration of reproducibility across observers and the broader applicability of the results in different clinical contexts.

Machining tests were carried out on the 3D-printed models. For the clinical validation of the materials, a questionnaire was prepared to evaluate the haptic sensation experienced during the machining tests on the surgical models and to compare it with that of real bones.

The statements were as follows:

- The feel of the trabecular bone is realistic.
- The feel of the cortical bone is realistic.
- The drilling is realistic.
- The milling/cutting operations are realistic.
- It is useful for simulating milling.
- It is useful for simulating drilling.
- It is useful for positioning screws or osteosynthesis materials.
- It is useful for simulating saw cutting.
- The behavior is similar to that of real bone.
- The model facilitates performing the different techniques.

Each statement was assessed using a scale from 1 to 5, with 1 being the lowest score mark and 5 being the highest.

## 3. Results

### 3.1. Melt Flow Index

The results of the MFI characterization for the two selected filaments are shown in Table 4.

The MFI results for similar filaments that were discarded are presented in Appendix A. Table A1 shows that, for PP + CaCO_3_, MFI decreases with increasing additive quantity. Consequently, the material becomes more viscous and more difficult to extrude as the filler quantity increases. This behavior can be attributed to the fact that higher filler content promotes particle–polymer and particle–particle interactions, leading to greater resistance to flow [28]. In contrast, for PP + GF filaments, the MFI increased with additive content, resulting in lower viscosity. Although fillers typically reduce MFI, the addition of glass fiber can degrade the PP matrix, for instance through chain scission caused by shear, heat, or catalyst residues during processing [29]. This degradation lowers the molecular weight of PP and may therefore increase the MFI.

### 3.2. Sterilization

The samples of the two selected materials (PP + 5% GF and PP + 35% CaCO_3_) successfully withstood the sterilization processes without any issues. To assess the condition of the tensile specimens, a visual inspection was carried out. The samples subjected to HPO sterilization were in good condition, displaying no visible deformations. The HPO process appeared to have minimal impact on the overall shape and integrity of the samples, suggesting its effectiveness in preserving their structural integrity.

On the other hand, the samples sterilized with Autoclave 121 and Autoclave 134 exhibited slight deformations at the specimens ends. Those deformations were likely caused by the high temperature and pressure applied during these sterilization processes. However, it is important to note that the deformations observed were relatively minor, indicating the overall structural integrity of the tensile strength specimens.

To qualitatively assess the deformation of the samples, the thickness values of the different specimens are presented in Table 5, both for the control samples and for those subjected to sterilization.

For glass fiber-filled polypropylene, the mean thickness after peroxide treatment was similar to that of the control samples. In contrast, the mean thickness of the specimens subjected to autoclave treatment was higher than that of the control samples. For calcium carbonate-filled polypropylene, the mean thickness of the samples increases after all three sterilization processes.

### 3.3. Characterization of Sterilized Samples

#### 3.3.1. PP + GF 5%

The results of the dimensional error, porosity, roughness, and maximum tensile strength characterization for PP + GF 5% are summarized in Table 6.

**Figure 7:**
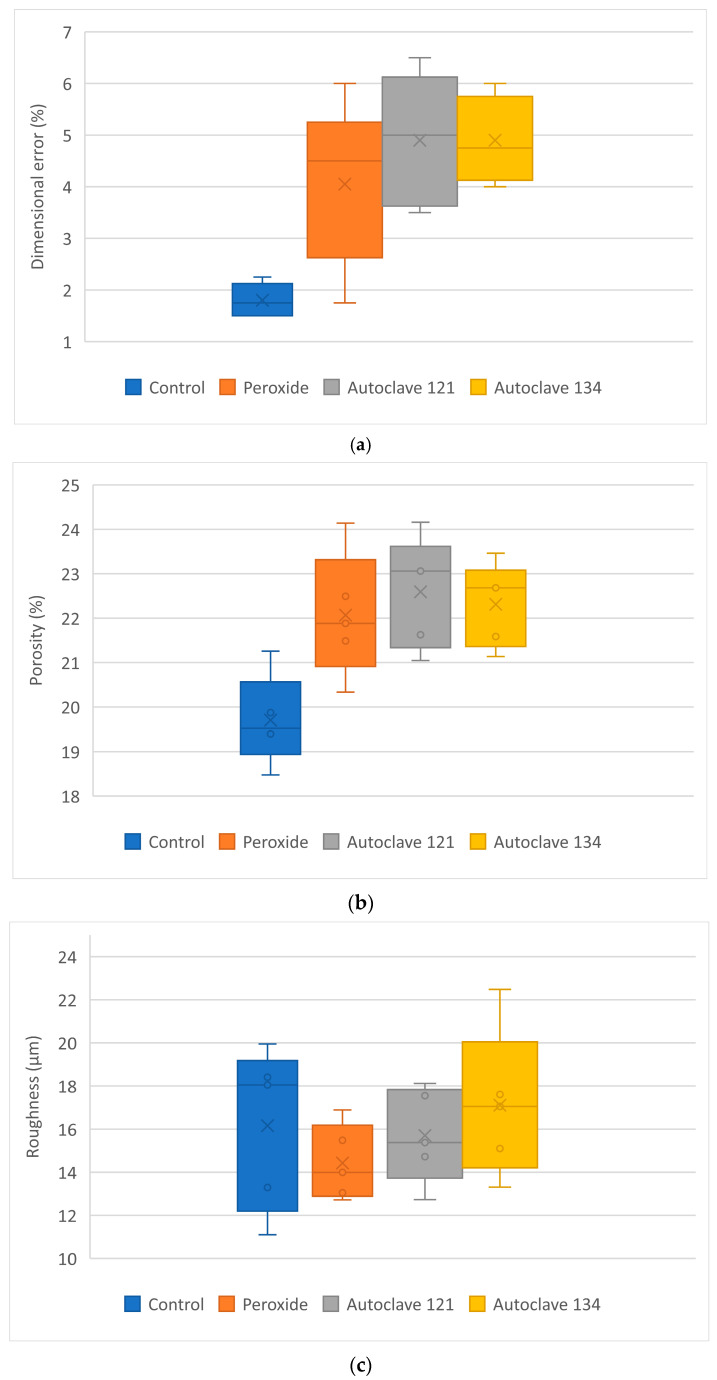

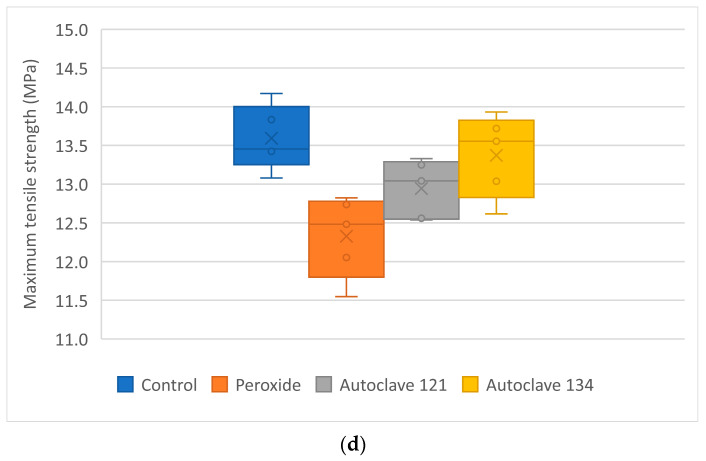
Characterization of PP + GF 5% samples: (**a**) dimensional error; (**b**) porosity; (**c**) roughness; (**d**) maximum tensile strength.

Figure 7 shows the box-plots for dimensional error, porosity, roughness, and maximum tensile strength.

As shown in Table 6 and Figure 7, the sterilized samples exhibited a higher dimensional error than the control samples. The mean dimensional error of the samples subjected to the three sterilization processes was similar, ranging between 4% and 5%, and higher than that of the control samples (around 1.80%). The *p* value for the control group versus any other group was <0.05, indicating significant differences. However, pairwise comparisons among the different sterilization treatments yielded *p* > 0.05, showing no significant differences between them.

The results show that the sterilized samples exhibited higher porosity levels compared with the control samples. This finding is consistent with the increase in the dimensional error of the samples. The *p* value for the control group versus any other group is <0.05, indicating significant differences. However, pairwise comparisons among the different sterilization treatments yielded *p* > 0.05, showing no significant differences between them.

The mean roughness values of the sterilized samples were similar to those of the control group, and the box plots did not reveal significant differences among the groups. All *p* values were >0.05, showing no statistically significant differences in roughness.

The mean maximum tensile strength of the control samples (13.59 MPa) was slightly lower than that of the PP raw material (15 MPa; Table 2). The maximum tensile strength of samples subjected to peroxide and autoclaving at 121 °C was significantly lower than that of the control samples, whereas the samples sterilized at 134 °C exhibited values similar to those of the control group. For the control and peroxide groups, *p* < 0.05, indicating significant differences between them. Pairwise comparisons among the other groups yielded *p* > 0.05, showing no significant differences.

#### 3.3.2. PP + CaCO_3_ 35%

The results regarding the characterization of dimensional error, porosity, roughness, and maximum tensile strength for PP + CaCO_3_ 35% are summarized in Table 7.

**Figure 8:**
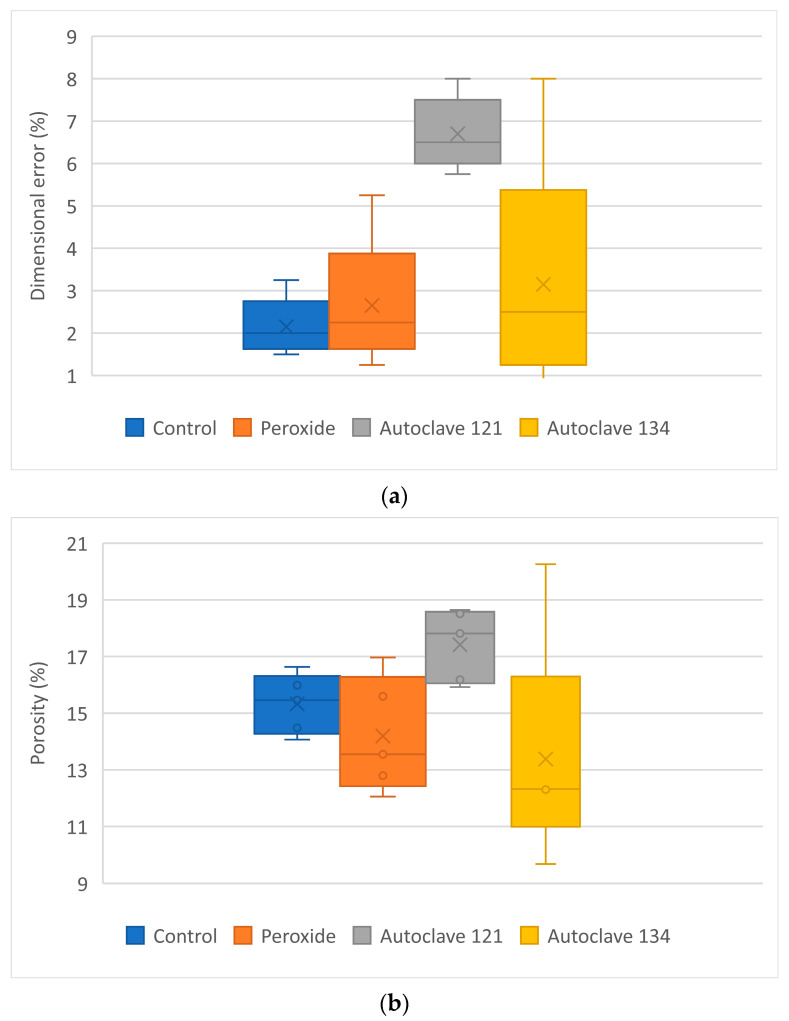

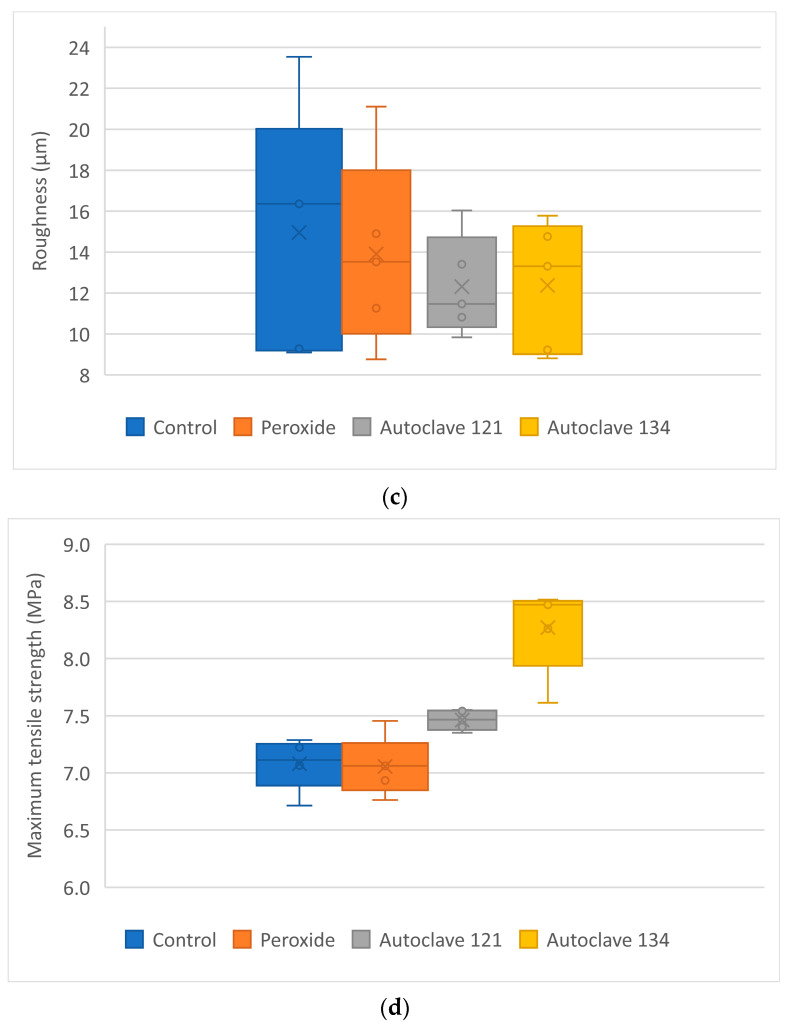
Characterization of PP + CaCO_3_ 35% samples: (**a**) dimensional error; (**b**) porosity; (**c**) roughness; (**d**) maximum tensile strength.

Figure 8 shows the box plots for dimensional error, porosity, roughness, and maximum tensile strength.

For the PP + 35% CaCO_3_ samples (Table 7 and Figure 8), those sterilized by autoclaving at 121 °C exhibited a higher dimensional error compared with the control, HPO, and autoclaving at 134 °C samples.

Comparisons between the Autoclave 121 group and any of the other groups yielded *p* < 0.05 (*p* = 0.0079), indicating significant differences. Pairwise comparisons among the remaining groups showed no significant differences (*p* > 0.05). For instance, the comparison between Autoclave 121 and Autoclave 134 resulted in *p* = 0.079.

Examining the porosity results, the samples sterilized by Autoclave 121 exhibited higher porosity than the other groups, consistent with the increased dimensional error. For porosity, comparisons between the Autoclave 121 group and each of the other groups yielded *p* < 0.05, indicating significant differences.

The roughness of the samples did not vary significantly with the sterilization treatments, although lower variability was observed compared with the control group. All *p* values were greater than 0.05.

The mean maximum tensile strength of the control samples of PP + CaCO_3_ 35% was approximately 7 MPa, considerably lower than that of the PP raw material. The maximum tensile strength increased after autoclaving, particularly at 134 °C, where it exceeded 8 MPa. This increase could be attributed to the formation of new compounds that may strengthen the samples. The effect of steam on the sample composition will be addressed in future research. Comparisons between the Autoclave 134 group and each of the other groups yielded *p* < 0.05, indicating significant differences. Additionally, the Autoclave 121 group also differed significantly from the control group.

### 3.4. Clinical Validation

**Figure 9:**
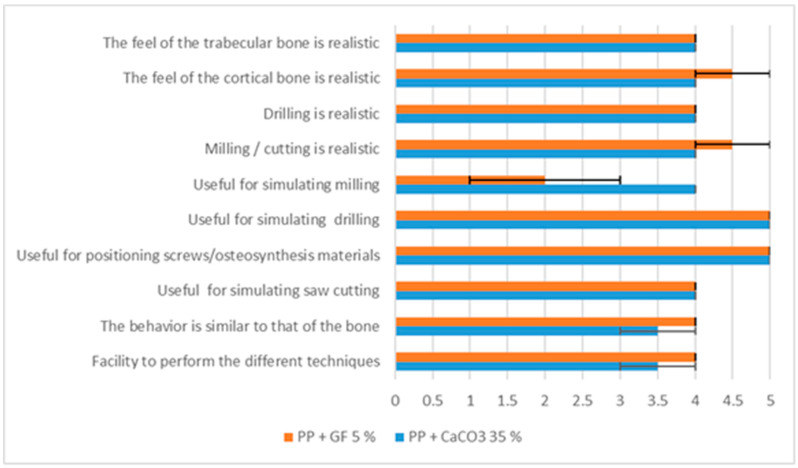
Results of the clinical validation of the mandible and femur models printed with PP + 35% CaCO_3_ and with PP + 5% GF (mean score of the two surgeons).

Clinical validation was successfully carried out in all cases. The models enabled the practice of sawing, milling, and the placement of plates. Figure 9 summarizes the results for all questions, showing the mean values and the error margins of the scores assigned by the surgeons.

Both materials received high scores, showing similar overall results. However, for question 5, which asked whether the materials were useful for simulating milling, the results differed: PP + 5% GF scored 2 and PP + 35% CaCO_3_ scored 4, indicating that the latter was more suitable for this purpose.

**Figure 10:**
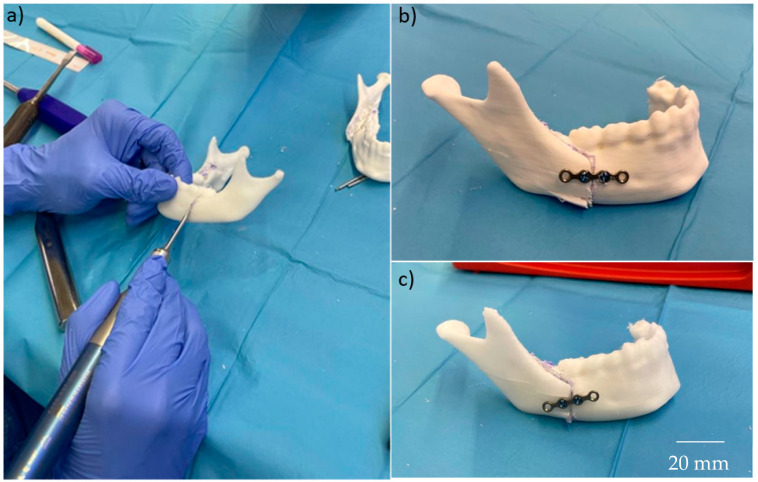
Results of the clinical validation for mandible samples. (**a**) Cutting process of a mandible with a saw. (**b**) Final result of the PP + 35% CaCO_3_. (**c**) Final result of the PP + 5% GF.

**Figure 11:**
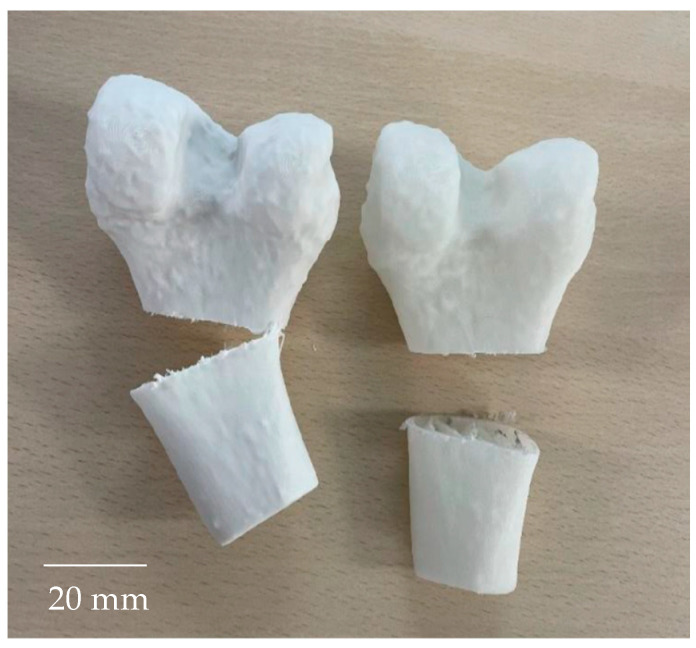
Results of the clinical validation for femur samples (PP + 35% CaCO_3_ and PP + 5% GF).

During the clinical validation, both models allowed for the positioning of mandibular plates, replicating a real surgical scenario, as shown in Figure 10. Similarly, both femur samples enabled successful for saw cutting (Figure 11).

In previous studies on cutting operations in pig mandible bones, irregular cuts in depth and shape were observed when metallic tools were used, specifically a high-speed handpiece (400,000 min^−1^) with a Lindemann bur, resulting in minimal thermal damage. When low-speed tools (up to 40,000 min^−1^) with round stainless-steel burs were used, deep and well-defined incisions were reported, but with thermal damage and some bone fragments [30]. As seen in Figure 11, similar irregular cuts and low thermal damage were observed in the present study.

## 4. Discussion

Nowadays, one of the most used 3D printing technologies for bone anatomical models is FFF. However, materials such as PLA and ABS still present limitations in mimicking bone properties and, in many cases, do not meet the required conditions for surgical simulations and training, including drilling, milling, and the placement of screws and surgical plates. Furthermore, materials used for surgical planning must withstand sterilization processes involving high temperatures and pressures, which can, in some cases, alter the final properties and geometry of the 3D-printed models.

In this study, six new PP-based formulated materials were developed and investigated. Two of these, PP + 35% CaCO_3_ and PP + 5% GF, were selected for further testing under different various conditions.

The analysis of the results highlights several key findings. Sterilized specimens exhibited higher dimensional errors than the control samples, with mean values reaching up to 6.70% for PP + 35% CaCO_3_ and up to 4.9% for PP + 5% GF, emphasizing the impact of sterilization procedures—particularly autoclaving—on the dimensional stability of the samples. These values are comparable to those reported by Popescu et al. [31] for ABS, which showed dimensional deviations of up to 9.33% after sterilization at temperatures above 60 °C. The observed variations in porosity among the samples corresponded to their dimensional increases after the sterilization process. In contrast, roughness did change vary significantly following the sterilization tests.

Maximum tensile strength did not vary significantly with the sterilization process, except for PP + 35% CaCO_3_, which showed a slight increase after autoclaving at 134 °C. A similar effect was reported by Fuentes et al. [32] in PLA samples, where moist heat sterilization increased crystallinity, leading to higher tensile modulus and impact resistance. Serbetci et al. [15] also observed an increase in maximum load before rupture values for PP meshes after a single autoclaving cycle, although this value decreased with subsequent cycles. When comparing the mechanical properties of the two new materials presented in this study (Table 5) with those of six commercially available filaments used in FFF printing (Table 2), it was observed that the tensile strength of the calcium carbonate-filled material closely resembled that of trabecular bone (ranging from 7 to 8.5 MPa). In contrast, the glass fiber-filled material exhibited a slightly higher tensile strength, up to 13.59 MPa, but remained lower than that of cortical bone and the commercial filaments.

Although their mechanical properties differ significantly from those of cortical bone, the milling, drilling, and cutting behavior of these materials effectively mimics bone, allowing surgeons to practice surgical skills. These materials may offer advantages over current alternatives, such as the Stratasys J850 Digital Anatomy, by reducing costs while replicating bone behavior. Evidence on the cost difference between 3D printing technologies has been reported by Chen et al. [33], showing that an orthopedic model printed with the Stratasys J750 Digital Anatomy ranges from 180.02 EUR to 255.21 EUR, whereas FDM reduces the cost to an average of 2.61 EUR.

In clinical settings, where 3D-printed anatomical models are increasingly used, it is crucial to understand the morphological and mechanical changes that may occur due to sterilization. Among the materials examined, hydrogen peroxide (HPO) appears to be the most suitable sterilization method, as the characterization results most closely resembled those of the control samples. These findings are consistent with reports from various authors [34,35,36].

The results of this study provide insights into the effects of different sterilization processes on the properties of the tensile specimens. They emphasize the importance of considering the potential impact of temperature and pressure during sterilization, particularly when using autoclave methods. Overall, these findings enhance the understanding of how sterilization affects the materials studied and underscore the need to select appropriate sterilization methods to minimize alterations in material properties for clinical applications. Both materials represent a viable alternative to currently used bone-modeling materials for surgical planning, although the maximum tensile strength of both 3D-printed materials remains far below that of cortical bone. Nevertheless, challenges remain, particularly in achieving a complete replication of bone’s hierarchical structure and complex biomechanics.

Future work will focus on biocompatibility testing as well as further development and validation of the best-performing materials. For the latter, additional testing will be performed with larger sample sizes and across a broader range of applications. The selected filaments will be evaluated using different 3D printers from multiple providers. Moreover, validation will be extended to new clinical applications and a wider variety of bones, including long, short, flat, and irregular bones, as well as different anatomical locations.

Although the current results are promising and provide initial guidance for identifying the most suitable materials for future studies, more extensive tests with a larger group of surgeons will be necessary to refine and validate their use in surgical applications. Furthermore, optimization strategies—such as fiber reinforcement, polymer blends, or surface coatings—will be explored to more closely replicate the mechanical properties of bone.

With regard to biocompatibility, although many surgical guides and anatomical models are limited to preoperative planning, training, or education, certain applications may require intraoperative use within the patient. In these cases, biocompatibility must be thoroughly assessed. According to ISO 10993-1 [37], this evaluation would involve cytotoxicity testing (to assess potential toxicity to mammalian cells), sensitization testing (to detect possible allergic reactions after repeated exposure), irritation/intracutaneous reactivity testing (to assess local effects on skin, mucosa, or subcutaneous tissue), and acute systemic toxicity testing (to determine whether leachables from the material produce harmful systemic effects after short-term exposure).

## 5. Conclusions

This study investigated the potential of six newly developed FFF materials for producing bone anatomical models intended for use as surgical planning simulators. The filaments were analyzed for their flow and printability behavior, with the best results obtained for two materials: PP + GF 5% and PP + CaCO_3_ 35%. These filaments were found to be easier to extrude and print than the other developed filaments.

After undergoing three different sterilization processes, it was found that dimensional error and porosity increased, while surface roughness does not change significantly. Tensile strength increased slightly for PP + CaCO_3_ when autoclaving at 134 °C was applied, whereas PP + glass fiber showed no change in tensile strength compared with the control samples.

The results underscore the importance of considering the effects of sterilization processes on the dimensional stability, porosity, roughness, and mechanical strength of these 3D-printed materials. These findings provide valuable guidance for selecting appropriate sterilization methods in clinical applications of the two materials.

Although biocompatibility is not crucial in this study, as the materials are intended for printing anatomical models for surgical practice, it will be addressed in future work to expand the potential applications of the materials.

## Figures and Tables

**Table 1:** Properties of cortical and trabecular bone.

	Compressive Strength (MPa)	Compressive Young’s Modulus (MPa)	Tensile Strength (MPa)	Tensile Young’s Modulus (MPa)	Flexural Strength (MPa)	Flexural Young’s Modulus (MPa)	Torsion Strength (MPa)	Torsion Young’s Modulus (MPa)
Cortical bone	167–231	14,700–34,300	107–170	11,400–29,200	103–238	9800–15,700	65–71	3100–3700
Trabecular bone	0.820–9.788	513–598	1.460–9.089	186.1–1759	-	-	0.297–5.448	17.46–358.2

**Table 2:** Properties of different FFF materials.

Material	Supplier	Melting Temperature (°C)	Tensile Strength (MPa)	Tensile Modulus (MPa)	Flexural Strength (MPa)
Polycarbonate (PC)	Innovatefil	250	65	2000	90
Polyoxymethylene (POM)	Ensinger	166	63	2600	83
Polylactide (PLA)	Smartfil	205	55.5	4635.7	107
Acrylonitrile Butadiene Styrene (ABS)	Smartfil	230	32.9	2300	65
High-Impact Polystyrene (HIPS)	Smartfil	230	12.9	1208.9	34.3
Polypropylene (PP)	LyondellBasell	230	15	800	950

**Table 3:** Printing settings for the tensile strength specimens.

	PP + GF 5%	PP + CaCO_3_ 35%
Nozzle diameter (mm)	0.4	0.4
Layer height (mm)	0.2	0.15
Printing temperature (°C)	240	250
Build plate temperature (°C)	30	30
Printing speed (mm/s)	30	40
Infill (%)	80	80
Infill pattern	Linear (0°, 90°)	Linear (0°, 90°)
Number of shells	3	3

**Table 4:** MFI results for the two selected filaments.

Material	Extruded Mass (g)	MFI (g/10 min)	Estimated Molecular Weight (g/mol)
PP + 35% CaCO_3_	2.15	6.387 ± 0.177	47,456.52
PP + 5% GF	1.63	9.805 ± 0.296	38,428.79

**Table 5:** Thickness values for the different samples (mm). Data is presented as mean ± STD.

	Group	Sample 1	Sample 2	Sample 3	Sample 4	Sample 5	Mean ± SD
PP + GF 5%	Control	4.06	4.09	4.08	4.06	4.07	4.07 ± 0.01
	Peroxide HPO	4.18	4.18	4.14	4.24	4.07	4.16 ± 0.06
	Autoclave 121	4.23	4.14	4.26	4.15	4.20	4.20 ± 0.05
	Autoclave 134	4.19	4.24	4.16	4.17	4.22	4.20 ± 0.03
PP + CaCO_3_ 35%	Control	4.13	4.06	4.07	4.09	4.08	4.09 ± 0.03
	Peroxide HPO	4.05	4.10	4.08	4.09	4.10	4.08 ± 0.02
	Autoclave 121	4.26	4.32	4.23	4.28	4.25	4.27 ± 0.03
	Autoclave 134	4.01	4.11	4.10	4.09	4.32	4.13 ± 0.12

**Table 6:** Characterization of PP + GF 5% samples. Data is presented as mean ± STD.

	Group	Dimensional Error (%)	Porosity (%)	Roughness (µm)	Maximum Tensile Strength (MPa)
PP + GF 5%	Control	1.80 ± 0.33	19.71 ± 1.01	16.15 ± 3.77	13.59 ± 0.42
	Peroxide HPO	4.05 ± 1.57	22.07 ± 1.40	14.43 ± 1.74	13.33 ± 0.53
	Autoclave 121	4.90 ± 1.28	22.59 ± 1.24	15.70 ± 2.19	12.94 ± 0.37
	Autoclave 134	4.90 ± 0.84	22.31 ± 0.94	17.11 ± 3.45	13.37 ± 0.54

**Table 7:** Characterization of the PP + CaCO_3_ 35% samples. Data is presented as mean ± STD.

	Group	Dimensional Error (%)	Porosity (%)	Roughness (µm)	Tensile Strength (MPa)
PP + CaCO_3_ 35%	Control	2.15 ± 0.68	15.33 ± 1.6	14.96 ± 6.01	7.08 ± 0.22
	Peroxide HPO	2.10 ± 0.52	14.19 ± 2.04	13.91 ± 4.65	7.06 ± 0.25
	Autoclave 121	6.70 ± 0.85	17.41 ± 1.29	12.32 ± 2.46	7.46 ± 0.09
	Autoclave 134	3.88 ± 2.76	13.38 ± 4.01	12.38 ± 3.19	8.27 ± 0.38

## Data Availability

The original contributions presented in this study are included in the article. Further inquiries can be directed to the corresponding author.

## Abbreviations

The following abbreviations are used in this manuscript:

ABS	Acrylonytrile Butadiene Styrene
AM	Additive manufacturing
BJ	Binder jetting
FDM	Fused deposition modeling
FFF	Fused filament fabrication
HIPS	High-impact polystyrene
HSJD	Sant Joan de Déu Barcelona Children’s Hospital
MEX	Material extrusion
MFI	Melt Flow Index
MJ	Material jetting
PBF	Powder bed fusion
PC	Polycarbonate
PLA	Polylactic acid
POM	Polyoxymethylene
PP	Polypropylene
SLA	Stereolithography
VPP	Vat photopolymerization

## Appendix A

Table A1 contains the MFI values for different filaments that were discarded for 3D printing.

**Table A1:** MFI results for the different filaments produced.

Material	Extruded Mass (g)	MFI (g/10 min)	Estimated Mw (g/mol)
PP + 50% CaCO_3_	2.60	4.713 ± 0.166	55,199.84
PP + 35% CaCO_3_	2.15	6.387 ± 0.177	47,456.52
PP + 15% GF	1.76	14.651 ± 0.213	38,428.79
PP + 10% GF	1.70	12.493 ± 0.201	36,344.79
PP + 8% GF	1.67	10.984 ± 0.204	33,899.47
PP + 5% GF	1.63	9.805 ± 0.296	31,178.57
